# Early Deregulation of Cholangiocyte NR0B2 During Primary Sclerosing Cholangitis

**DOI:** 10.1016/j.gastha.2022.07.023

**Published:** 2022-08-13

**Authors:** Christophe Desterke, Chuhan Chung, David Pan, Michael Trauner, Didier Samuel, Daniel Azoulay, Cyrille Feray

**Affiliations:** 1 Hôpital Paul-Brousse, Institut National de la Santé et de la Recherche Médicale UMRS1310, Université Paris-Saclay, Villejuif, France; 2 Gilead Sciences, Inc, Foster City, California; 3 Division of Gastroenterology and Hepatology, Department of Medicine III, Medical University of Vienna, Vienna, Austria; 4 Centre Hépato-Biliaire, Hôpital Paul-Brousse, Assistance Publique-Hôpitaux de Paris, Institut National de la Santé et de la Recherche Médicale U1193, Université Paris-Saclay, Villejuif, France

**Keywords:** Bile Acid Metabolism, Text Mining, Premalignancy, Transcriptome

## Abstract

**Background and Aims:**

Primary sclerosing cholangitis (PSC) is a probable autoimmune liver disease characterized by persistent and progressive biliary inflammation that leads to biliary infection, cirrhosis, or cholangiocarcinoma. Genome-wide omics data are scarce regarding this severe disease.

**Methods:**

MEDLINE database gene prioritization by text mining (biliary inflammation, biliary fibrosis, biliary stasis) was integrated in distinct omics data: (1) PSC liver transcriptome training and validation cohorts, (2) farnesoid X receptor (FXR) mice liver transcriptome subjected to an FXR agonist or FXR knockout mice; (3) liver single-cell transcriptome of the Abcb4−/− mice model of PSC.

**Results:**

A liver molecular network highlighted the involvement of nuclear receptor subfamily 0 group B member 2 (NR0B2) and its associated nuclear receptor FXR in a metabolic cascade that may influence the immune response. NR0B2 upregulation in PSC liver was independent of gender, age, body mass index, liver fibrosis, and PSC complications. Heterogeneity of NR0B2 upregulation was found in cholangiocyte cell types in which the NR0B2-based cell fate decision revealed the involvement of several metabolic pathways for detoxification (sulfur, glutathione derivative, and monocarboxylic acid metabolisms). Genes potentially implicated in carcinogenesis were also discovered on this cholangiocyte trajectory: GSTA3, inhibitor of DNA binding 2, and above all, TMEM45A, a transmembrane molecule from the Golgi apparatus considered as oncogenic in several cancers.

**Conclusion:**

By revisiting PSC through PubMed data mining, we evidenced the early cholangiocyte deregulation of NR0B2, highlighting a metabolic and premalignant reprogramming of the cholangiocyte cell type. The therapeutic targeting of NR0B2 could potentiate that of FXR and enable action on early events of the disease and prevent its progression.

## Introduction

Primary sclerosing cholangitis (PSC) is a progressive, cholestatic liver disease characterized by gradual inflammation, destruction, and fibrosis of the intrahepatic and extrahepatic bile ducts.^1^ The incidence of PSC ranges from 0 to 1.3 cases per 100,000 persons per year.^2^ Concomitant inflammatory bowel disease (IBD), most frequently ulcerative colitis, has been reported in up to 80% of patients with PSC.^3^ The close association between PSC and IBD indicates involvement of the gut-liver axis. Environmental factors such as the microbiota have also emerged as potential players in chronic inflammatory diseases like PSC.^4^ The enterohepatic circulation may be important in PSC.^5^ Although classically seen as an autoimmune disease, the pathogenesis of PSC remains unclear. Very few transcriptomic studies are available in PSC patients.^6^^,^^7^ Biliary cirrhosis, bacterial cholangitis, and cholangiocarcinoma are the principal complications. No effective treatment is yet available although positive results have been published following the use of obeticholic acid, a potent farnesoid X receptor (FXR) agonist.^8^

Among the different biomedical text mining tools designed to link keywords with omics data, the “Génie” algorithm^9^ offers a robust and sensitive machine learning tool. By text mining (Génie) integration in the single-cell transcriptome of a kidney data set, we were able to highlight podocyte markers implicated in focal segmental glomerulosclerosis.^10^ The “Génie” algorithm can also help to interpret deregulations that occur during nonalcoholic fatty liver disease (NAFLD).^11^ “Génie” is a tool that prioritizes whole gene sets of hundreds of species for any biomedical topic, taking advantage of gene identifier links with the bibliography.^9^ During the present work, biomedical text mining related to PSC phenotypes enabled the definition of a gene set that discriminates omics data on human PSC liver biopsies and distinct animal models of PSC. The independent deregulation of nuclear receptor subfamily 0 group B member 2 (NR0B2) in PSC was able to highlight metabolic and premalignant reprogramming in cholangiocyte cell types.

## Results

### Biomedical Text Mining to Discover Genes Related to PSC

**Figure 1:**
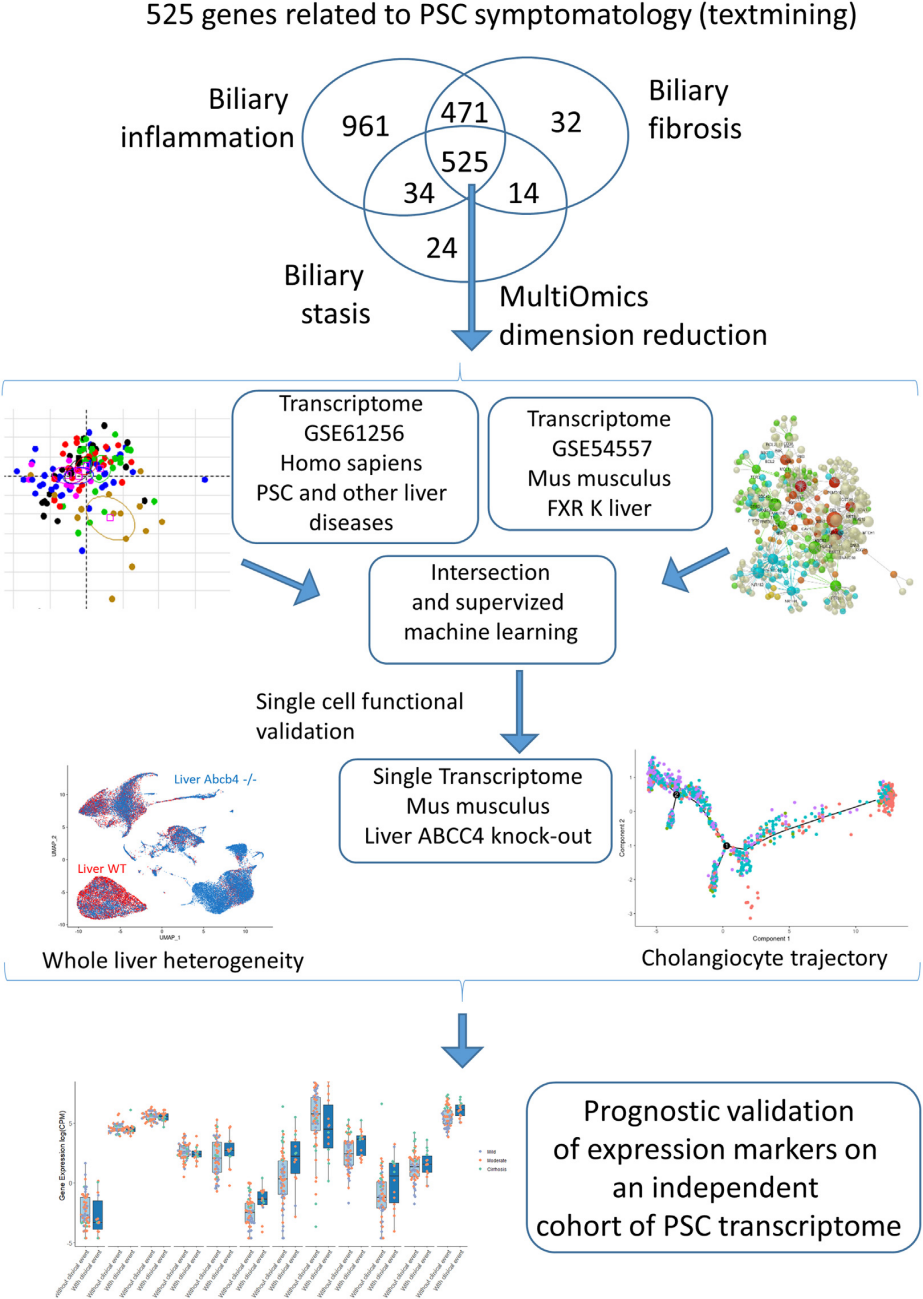
Workflow of bioinformatics analyses. This figure describes the workflow of the bioinformatics process performed on the data used in this work.

Text mining gene prioritization was performed by querying the MEDLINE database^12^ based on keywords associated with PSC^1^ (Figure 1). The Génie algorithm enables the retrieval of all literature attached to all genes in a genome and to their orthologs according to a selected topic.^13^ It is based on Naive Bayes classifiers and has the advantage of including the genes of different species.^9^ Following the review by Lazaridis and LaRusso,^1^ 3 Medical Subject Heading terms for the characterization of PSC were used in the “Génie” algorithm: “biliary inflammation”, “biliary fibrosis”, and “biliary stasis”, giving 3 lists of genes. The intersection of these 3 gene sets gave rise to a list of 525 ranked genes (workflow, Figure 1 and Table A1). NR1H4 (alias FXR) was the top-ranked gene (Table A1). This set of 525 PSC-related genes was used to investigate downstream omics analyses in the context of PSC disorders, as shown in the workflow (Figure 1). Starting the omics analyses with reduced-size gene sets enabled a reduction in background noise and the false discovery rate (FDR) during these experiments, as has been shown with the Gene Set Enrichment Analysis procedure.^14^

### Validation of PSC-Related Genes

**Figure 2:**
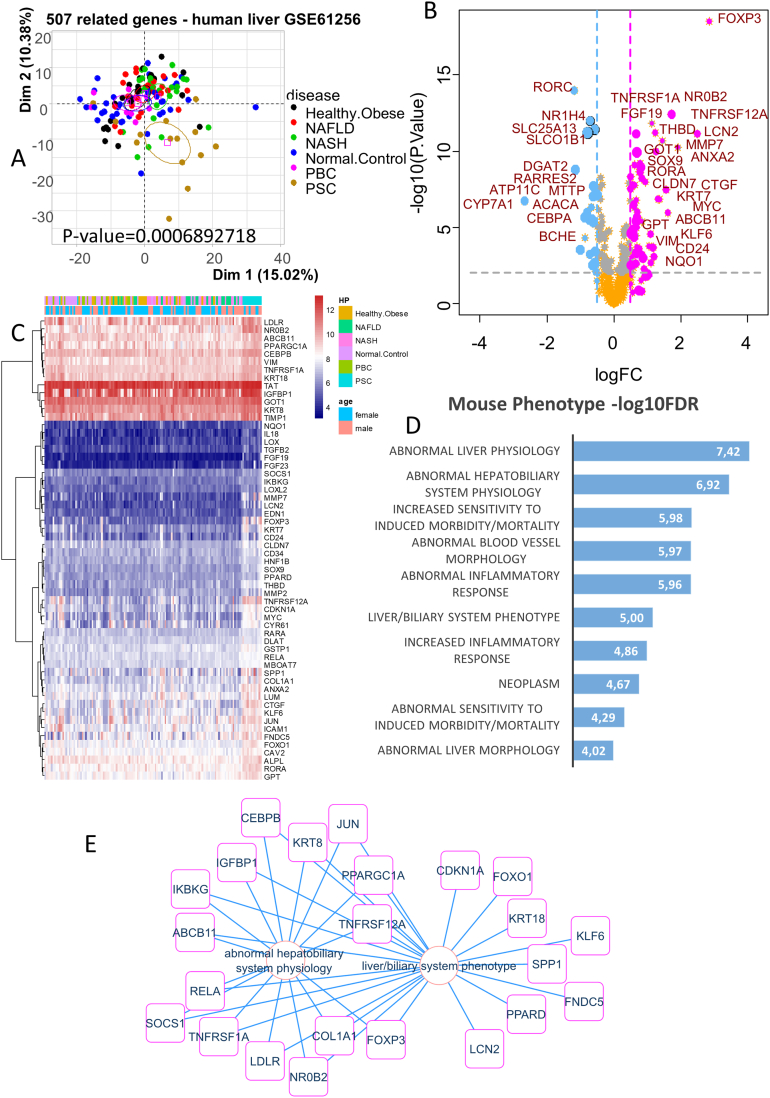
Abnormal hepatobiliary system physiology in liver biopsies from primary sclerosing cholangitis patients. Investigations on transcriptome data set GSE61256: (A) principal component analysis performed on 507 detected symptom-related genes on liver biopsy transcriptomes from distinct groups of diseases and controls; NASH, nonalcoholic steatohepatitis. (B) Volcano plot of differentially expressed genes (DEGs) between PSC liver and other liver biopsies. (C) Expression heatmap of significantly upregulated genes in PSC liver samples. (D) Functional enrichment performed on the Mouse Phenotype database with genes upregulated in PSC liver samples. (E) Functional enrichment network with PSC upregulated genes implicated in the hepatobiliary mouse phenotype.

We tested the set of PSC-related genes on the transcriptome study GSE61256^7^ (Figure 1) which included liver biopsy samples from patients with PSC (n = 14) or primary biliary cholangitis (PBC) (n = 11), obesity (n = 24), NAFLD (n = 23), nonalcoholic steatohepatitis (n = 24), and normal controls (n = 38). This data set was not found using the following 3 keywords: “biliary inflammation”, “biliary fibrosis”, and “biliary stasis” in the Gene Expression Omnibus database. After gene annotation with the corresponding platform, data from GSE61256 were reduced in dimension with 507 of 525 presenting PSC-related genes. Unsupervised principal component analysis revealed a clear clustering of PSC samples as compared to the others and particularly compared to PBC, a disease that affects the small biliary ducts (*P* value = 6.89E-4, Figure 2A). However, these genes discriminated PBC samples from others in the second map (PCA dimensions 1 and 3) of the PCA analysis (Figure A1). Taken together, this suggested that the PSC-related genes highlighted by Genie text mining were relevant. In Table 1, we show the top 10 regulated genes, the first being FXR.

**Table 1:** Best 10 Markers of PSC Ranked by Text Mining: For Respective Gene Columns Showing the Text Mining Rank and FDR-Adjusted *P* Value for 3 Symptoms (Biliary Fibrosis, Biliary Inflammation, Biliary Stasis)

Gene	Rank biliary fibrosis	Rank biliary inflammation	Rank biliary stasis	FDR biliary fibrosis	FDR biliary inflammation	FDR biliary stasis
NR1H4	1	3	1	7.785e-144	4.838e-210	6.278e-212
ABCB4	4	10	3	1.455e-118	2.618e-127	7.063e-161
ABCB11	5	18	2	6.742e-98	8.01e-106	2.523e-176
TGFB1	6	8	19	1.35e-79	8.031e-138	5.692e-40
IFNL3	2	27	7	5.873e-129	1.062e-87	7.138e-79
PNPLA3	3	42	6	3.532e-128	8.692e-70	1.284e-79
IL6	15	1	36	7.473e-45	1.318e-230	9.478e-27
TLR4	18	4	33	7.342e-43	1.276e-188	2.147e-27
GPBAR1	10	38	12	3.938e-54	1.874e-73	5.339e-52
IL17A	14	6	44	6.968e-45	1.34e-170	2.905e-26

Analysis of these 507 genes using the supervised “Linear Model from Microarray” algorithm provided the expression profile of PSC liver samples compared to all the other pooled control groups (Figure 2B and Table A2). *FOXP3* was the leading upregulated gene, followed by *NR0B2*, *TNFRSF1A*, and *FGF19*. A distinct trait of expression which was upregulated in PSC samples (cyan blue annotated samples) could be observed on the right-hand side of the expression heatmap (Figure 2C).

### Characterization of PSC-Related Genes in the Mouse Phenotype Database

During “Génie” algorithm text mining on PSC, the option of a gene paralog was implemented during the literature search as it could take advantage of combining results obtained in humans and in animal models. Scientific experiments performed on mice models have made a significant contribution to understanding the pathophysiology of PSC through bile acid (BA) homeostasis.^15^ Functional enrichment using upregulated PSC-related genes was performed on the Mouse Phenotype database and indicated an abnormal physiology of the liver and hepatobiliary system (Figure 2D). On the phenotype network for the hepatobiliary system, we were able to observe some of the leading genes upregulated in PSC such as *FOXP3*, *TNFRSF12A*, *TNFRSF1A*, *LCN2*, and *NR0B2* (Figure 2E). Starting from the PSC-related genes found by text mining, we were then able to define a PSC-specific upregulated network implicated in the hepatobiliary system phenotype.

### Hepatic Regulation of the Immune Response and Intracellular Receptor Signaling Under FXR Dependency

**Figure 3:**
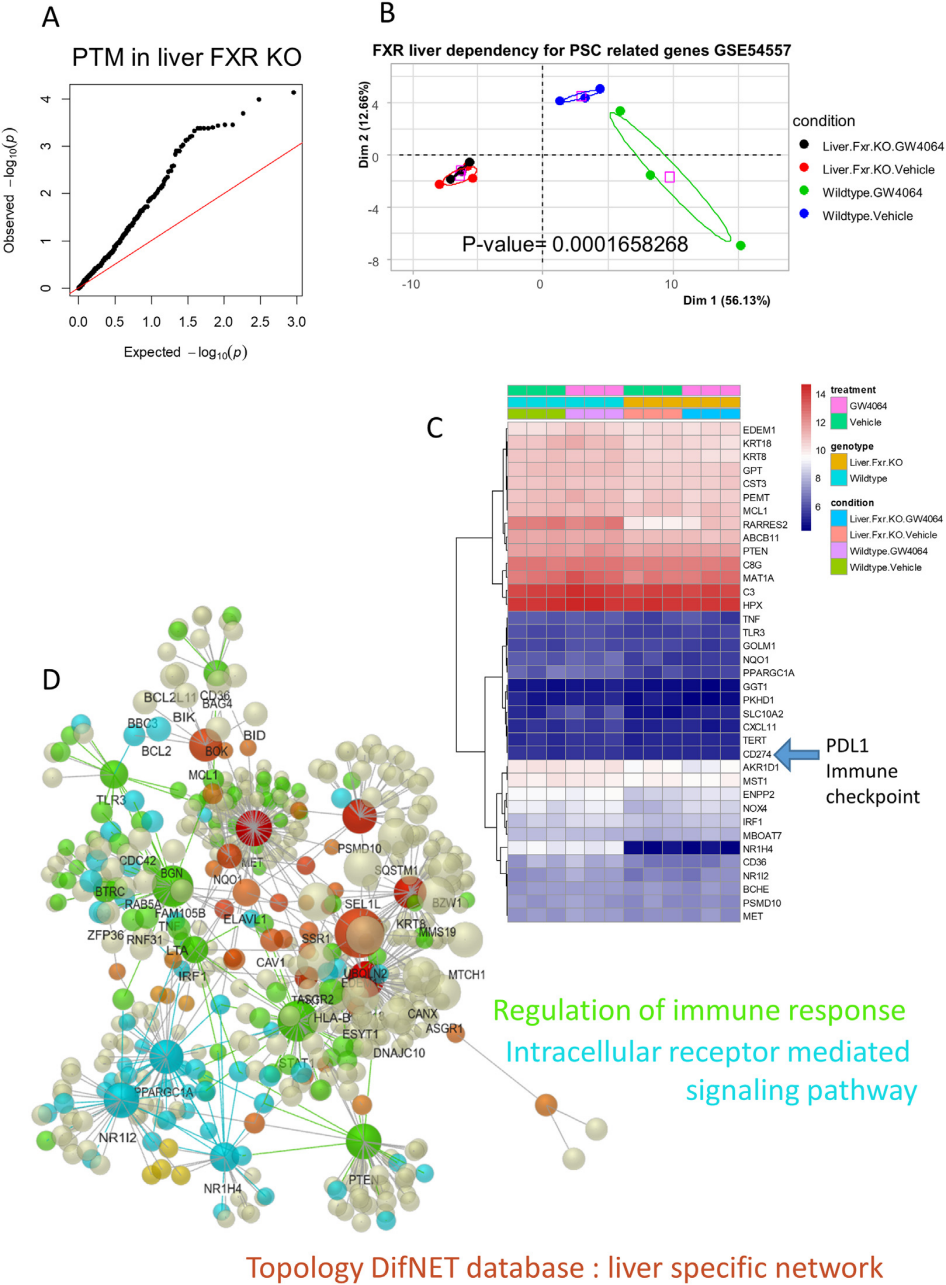
Hepatic regulation of the immune response and intracellular receptor signaling under FXR dependency. Investigations on transcriptome data set GSE54557: (A) qqplot of *P* values obtained by the Pavlidis Template Matching (PTM) algorithm with symptom-related genes for liver FXR regulation dependency. (B) Principal component analysis performed with PSC symptom-related genes under liver FXR regulation; GW4064, FXR agonist. (C) Expression heatmap for PSC symptom-related genes found to be positively correlated to hepatic FXR regulation. (D) Protein-protein interaction network based on PSC symptom-related genes upregulated in PSC liver samples with colored function inference of the Gene Ontology Biological Process (GO-BP) database; in green, enrichment of the immune response function, and in blue, intracellular receptor signaling function.

NR1H4 (or FXR) was the highest ranked (Table 1) among the PSC-related genes. FXR is a nuclear receptor (NR) implicated in BA metabolism.^16^ The GSE54557 transcriptome data set includes the gene expression of *Mus musculus* liver with or without liver FXR knockout (KO), reversed or not by FXR agonist treatment.^16^ Data from GSE61256 were reduced in dimension with the 525 PSC-related genes, and the Pavlidis Template Matching algorithm^17^ was used to analyze FXR-dependent regulations (Figure 3A and Table A3). Ninety-one genes from the 525 PSC-related genes were regulated through FXR in the mouse liver samples. In turn, this set of genes allowed stratification of the samples of FXR liver KO from wildtype samples (WT), and reversion by the FXR agonist was only obtained only in the latter (*P* value = 1.66.E-4, Figure 3B). Genes positively regulated by FXR were selected to draw an expression heatmap (Figure 3C) which involved a protein-protein interaction (PPI) network mainly implicated in the immune response (nodes in green, Figure 3D) and in the intracellular receptor-mediated signaling pathway (in blue, Figure 3D). Concerning the latter, 3 main blue crosstalk patterns could be observed on the PPI network (Figure 3D): NR1H4, NR1I2, and PPARGC1A were all closely connected. In the former case, we observed (Figure 3C) a liver FXR regulation dependency of CD274 (ie, the PDL1 immune checkpoint). Starting from the PSC-related gene list, we were able to predict a hepatic expression profile dependent on FXR regulation and thus confirmed its potential functional importance to the pathology.

### NR0B2 Was Regulated in the Livers of PSC Patients Under FXR Dependency

**Figure 4:**
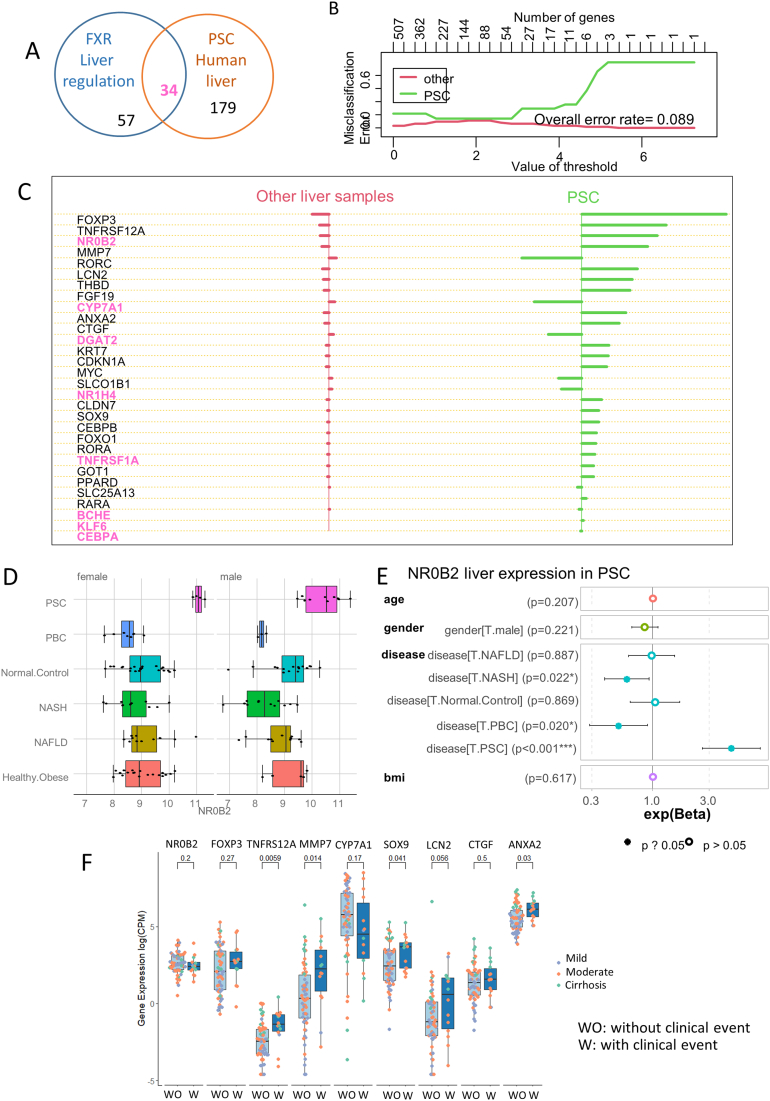
Independent of patient prognosis, NR0B2 was found to be regulated in the livers of those with primary sclerosing cholangitis under FXR dependency. (A) Venn diagram merging the FXR liver regulation dependency of PSC-related genes found to be upregulated in PSC human liver biopsies. (B) Supervised machine learning misclassification plot by class obtained with PSC symptom-related genes to stratified PSC liver samples taken from other control samples in GSE61256. (C) Ranked predictive scores obtained by machine learning on the GSE61256 data set with PSC symptom-related genes; the FXR dependency of hepatic regulation is highlighted in pink for the genes concerned. (D) Boxplot of the regulation of NR0B2 expression based on liver group samples and stratified on gender (GSE61256). (E) Multivariate model based on NR0B2 expression: exponential coefficients and their confidence intervals (95%) were drawn for each covariate. (F) Boxplot of the validation of expression of 9 liver PSC markers according to the clinical grades of patients in an independent validation cohort.

In order to evaluate FXR dependency in the PSC-related human liver expression profile, we then crossed the PSC human liver signature (GSE61256) (Table A2 and Figure 2) with the mouse liver FXR functional signature (GSE54557) (Table A3 and Figure 3). The overlaps of this analysis determined a set of 34 common genes (Figure 4A). A machine learning model based on leave-one-out cross-validation (pamr) was supervised between the human liver PSC samples and other liver samples (GSE61256) and, thus, define a predictive molecular ranking (Table A2) with a minimal misclassification error of 8.9% between group samples (Figure 4B). Within this optimum molecular ranking to predict human PSC liver, we observed 8 molecules subject to FXR-dependent regulation (in pink, Figure 4C), that is, NR0B2, CYP7A1, DGAT2, NR1H4 (alias FXR), TNFRSF1A, BCHE, KLF6, and CEBPA. At the top of the PSC prediction list regarding FXR dependency, NR0B2 was investigated by univariate analysis stratified on the gender of the patients; it was upregulated in PSC samples (Figure 4D), and this was independent of other covariates (Figure 4E).

### PSC Liver Markers in an Independent Cohort of PSC Transcriptomes With Clinical Annotations

Among the best predictive markers of PSC liver samples identified by machine learning (Figure 4C), we determined whether NR0B2 and a set of 8 other genes were related to the presence of fibrosis and PSC complications. The choice of these biomarkers for external validation was mainly driven by their upregulation found in PSC liver samples (Figure 4C) and also by their potential impact on PSC physiopathology through their molecular functions such as immunity (FOXP3, TNFRS12A, LCN2), microenvironment (MMP7), cell growth (ANXA2), and cholangiocyte markers (SOX9, CTGF). Furthermore, CYP7A1 was chosen for the validation of expression because this molecule is already known as a repressed target of FXR.^18^ NR0B2, the top-ranked upregulated and FXR-dependent molecule (Figure 4C) in PSC liver, was associated with neither PSC complications nor the degree of fibrosis (Figure 4F). As for CYP7A1, which was found to be repressed in PSC livers (Figure 4C), it had a tendency to be repressed in the context of PSC with an adverse clinical profile (*P* value = .17, Figure 4F). Among the other tested markers, complications of PSC were associated with the upregulation of TNFRS12A (ie, Tweak-receptor or CD266 [*P* value = .006]), SOX9 (*P* value = .041), ANXA2 (*P* value = .03), MMP7 (*P* value = .014), and LCN2 (*P* value = .05, Figure 4F).

### Upregulation of NR0B2 in Cholangiocytes From Abcb4−/− Livers

**Figure 5:**
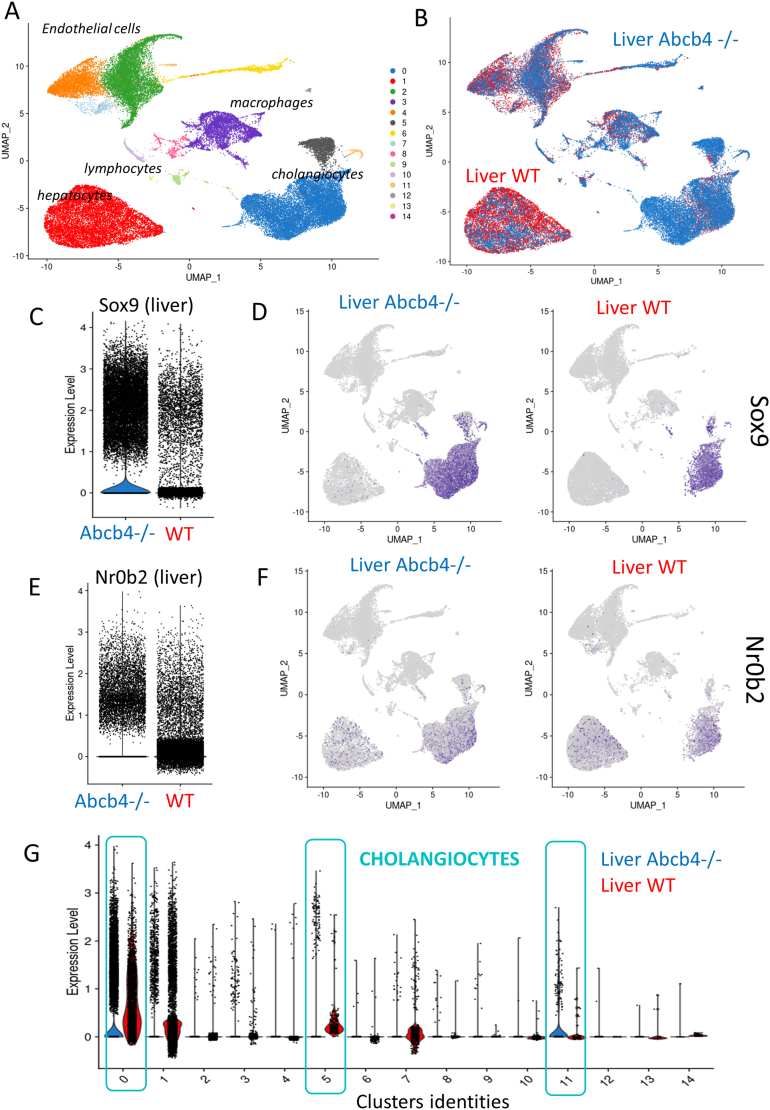
Upregulation of NR0B2 in the cholangiocyte compartment of Abcb4−/− livers. Investigations on the whole-liver single-cell transcriptome data set GSE168758. (A) Uniform Manifold Approximation and Projection (UMAP) dimension reduction with identification of the distinct cell compartments in whole liver samples: colors reflect 15 distinct cluster cell communities. (B) UMAP with genotype stratification: Abcb4−/−, Abcb4 knockout. (C) Violin plot of SOX9 expression in whole liver stratified on genotypes. (D) SOX9 feature plot stratified on genotypes, with blue for positive expression. (E) Violin plot of NR0B2 expression in whole liver stratified on genotypes. (F) NR0B2 feature plot stratified on genotypes, with blue for positive expression. (G) Violin plot of NR0B2 expression stratified on cluster cell communities and genotypes.

Adenosine triphosphate-binding cassette subfamily B member 4 (Abcb4)−/− (ie, Mdr2: multiple drug resistance 2) mice are used as a model for sclerosing cholangitis because they reflect the progressive fibrosing cholangitis aspects of human PSC and also develop intrahepatic cholangiocarcinoma.^19^^,^^20^ Abcb4 is an adenosine triphosphate-binding cassette transporter that is required for the biliary excretion of phosphatidylcholine.^21^ Single-cell transcriptomes from wild-type and Abcb4−/− mouse models are provided by the GSE168758 data set.^22^ With 4 replicates in each group, a single-cell WT and Abcb4−/− normalized object was built with 46,087 cells which individually expressed a minimum of 300 genes. After dimension reduction and clustering, 15 cell communities were identified and classified in 5 cell types: hepatocytes, cholangiocytes, endothelial cells, macrophages, and lymphocytes (Figure 5A and Figure A2). Abcb4−/− livers harbored more cholangiocytes than wild-type livers (in blue, Figure 5B). SOX9, a transcription factor involved in biliary development,^23^^,^^24^ was found to be overexpressed in Abcb4−/− livers when compared to wild-type livers (Figure 5C) and particularly in the cholangiocyte compartment (Figure 5D) which corresponds to cell clusters 0, 5, and 11 (Figure A3A). Similarly, an overexpression of NR0B2 was found in Abcb4−/− livers (Figure 5E). NR0B2 was detected in hepatocytes and cholangiocytes in both liver genotypes (Figure 5F). The overexpression of NR0B2 in Abcb4−/− livers concerned cell clusters 0, 5, and 11 (Figure 5G and Figure A3B) which also expressed SOX9 and Epcam (Figure A3, A and C). The absence of NR0B2 regulation was observed in the hepatocyte compartment of Abcb4−/− (cluster 1, Figure 5G). These results suggest that the NR0B2 NR was upregulated in the cholangiocytes of a mouse model of sclerosing cholangitis.

### Metabolic Regulation During the Cell Fate Decision of Abcb4−/− Cholangiocytes

**Figure 6:**
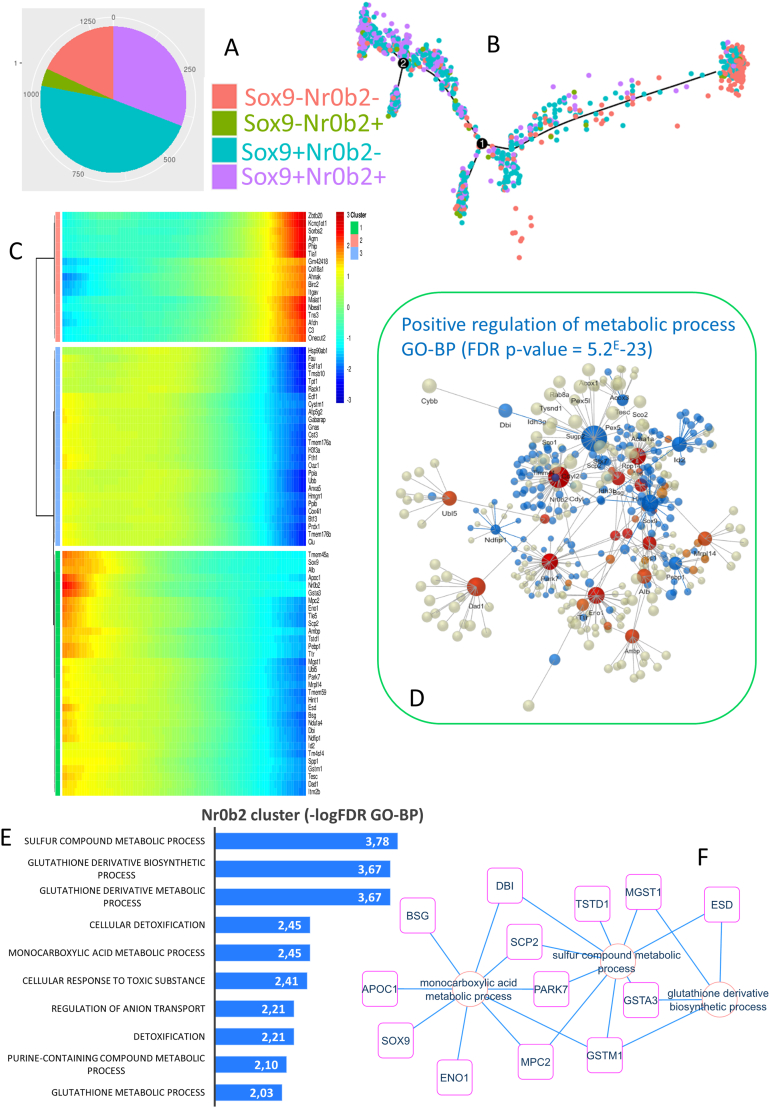
Metabolic regulation during cell trajectory of Abcb4−/− cholangiocytes. Sampling of cholangiocytes identified as Abcb4−/− in the liver single-cell transcriptome data set GSE168758: (A) Pie chart of cell stratification as a function of the alternative expression of NR0B2 and SOX9 in cholangiocytes from Abcb4−/− liver samples. (B) Abcb4−/− cholangiocyte single-cell trajectory identified from the alternative expression of NR0B2 and SOX9. (C) Pseudotime expression heatmap of genes found to be significantly regulated on the NR0B2-SOX9 single-cell trajectory of Abcb4−/− cholangiocytes: green clusters follow SOX9 and NR0B2 pseudotime expression. (D) Protein-protein interaction network identified on the pseudotime green cluster: Blue characterizes the network inference of positive regulation of the metabolic process. (E) Bar plot of functional enrichment performed on the pseudotime green cluster with the Gene Ontology Biological Process (GO-BP) database. (F) Functional enrichment network performed with the best metabolic function enriched on the pseudotime green cluster.

NR0B2 (ie, small heterodimer partner) is a transcription regulator involved in BA metabolism, lipid metabolism, and glucose and energy homeostasis.^25^ Starting from the single-cell transcriptome GSE168758 data set, a random subset of Abcb4−/− cholangiocytes was selected to build a single-cell trajectory based on the alternative expression of NR0B2 and SOX9. Within the cholangiocyte compartment of 1316 cells, 238 cells were SOX9- and NR0B2- double negative, 51 cells were SOX9- and NR0B2+, 406 cells were SOX9+ and NR0B2-, and 621 cells were SOX9+ and NR0B2+ double positive (Figure 6A and Figure A4, A and B). Pseudotime transformation of a single-cell transcriptome in this selected compartment (Figure 6 and Figure A4C) reflected a cell trajectory involving progressive modification of the expression of SOX9 (Figure A4D) and NR0B2 (Figure A4E). Identification of the most significant regulated genes on this cell trajectory (Table A4) revealed 3 regulated gene clusters (Figure 6C). The expressions of SOX9 and NR0B2 were closely regulated on the trajectory of the pseudotime green gene cluster (Figure 6C), where the expression of Alb, GSTA3, inhibitor of DNA binding 2 (ID2), and TMEM45A were found to be regulated alongside NR0B2 and SOX9 (Figure 6C and Figure A4F). A protein-protein network performed with genes belonging to the green pseudotime gene cluster with functional inference of the Gene Ontology Biological Process database was able to identify functional enrichment of the positive regulation of metabolism (FDR *P* value = 5.2E-23, Figure 6D) which mainly involved sulfur compound metabolism, glutathione derivative biosynthesis, cellular detoxification, and monocarboxylic acid metabolism (Figure 6E), all of which share certain main regulated molecules (Figure 6F). These results suggested that the heterogeneity of NR0B2 expression in Abcb4−/− cholangiocyte-affected compartments defined a cell trajectory that involved the important regulation of metabolism with the detoxification process.

## Discussion

The integration of omic-type biological data—either published or available from clinical, animal, and cellular models—enabled a synthesis of knowledge and the determination of genes or pathways that had not previously been highlighted. This is classic for genomic-type data and is becoming widespread in oncology through the use of the The Cancer Genome Atlas database. It is much less common in the field of physiopathology, particularly with regard to rare diseases for which omic data are rare. For example, through the text mining of lipid-related genes, we were able to link inflammation to hepatocarcinogenesis during nonalcoholic hepatitis.^26^ We used the “Génie” text mining program based on physiopathological keywords which delivers and ranks a set of genes cited in the PubMed, MEDLINE, and National Center for Biotechnology Information (NCBI) database^12^ and then cross-validated this data set with the relevant omic data.

Based on 3 keywords—biliary inflammation, biliary fibrosis, and biliary stasis^1^—525 genes were found to be related to PSC.^27^ This set of genes was challenged on liver RNA omics data from patients with diverse causes of liver disease (GSE61256). The paper from which this data set was derived was not found using the keywords because its subject was NAFLD, and PSC patients were only included in the control group.^7^ These genes showed a clear clustering of PSC samples. The top-ranked gene was NR1H4 (FXR) for which role/therapeutic target is clear in both cholestatic and fatty liver diseases.^28^ By analyzing these 525 genes in FXR KO mice and the liver single-cell transcriptomes of PSC Abcb4−/− mice, we were able to reveal the important involvement of NR0B2 which is both FXR-dependent at transcriptional level and a NR associated with FXR NRs and thus involved in numerous pathways such as metabolism, cell proliferation, and inflammation in BA metabolism.^29^ NR0B2 was upregulated in PSC livers independent of gender, age, and body mass index. Importantly, it was not dependent on the severity of PSC in the prognostic cohort, suggesting that this may be an early event during the disease.

Through liver functional analysis, FXR regulation dependency was used to build a liver PPI network which mainly implicated the immune response and NR signaling. Through the immune response functionality, CD274 (alias PDL1) was found to be regulated under liver FXR regulation. It should be noted that pembrolizumab, which is an anti-PDL1 drug, has been reported as a cause of sclerosing cholangitis.^30^

Concerning immune deregulations, the FOXP3 transcription factor was found overexpressed in the livers of PSC patients. FOXP3 characterizes the phenotype of the regulatory T-lymphocyte immune cell subpopulation.^31^ In PSC patients, a polymorphism found in the *IL2RA* gene was seen to affect FOXP3+ regulatory T cells,^32^ and it has been suggested that an imbalance between Foxp3+ regulatory T cells (Treg) and Th17 cells may be involved in IBD and PSC.^33^

As bulk transcriptomes capture an average transcriptome for all the cell types present in a biopsy, single-cell RNA sequencing permits identification of the cell type in which RNA is regulated. Abcb4−/− mice are widely used as a model of sclerosing cholangitis. At the single-cell level in the livers of Abcb4−/− mice, the expression of NR0B2 was detected in the hepatocyte and cholangiocyte cell compartments, but its overexpression was only observed in cholangiocytes that strongly expressed SOX9, which is a transcription factor involved in biliary development.^23^ Based on the alternative expression of SOX9 and NR0B2, a single-cell trajectory was built with the Abcb4−/− cholangiocyte compartment highlighting a network of molecules implicated in the metabolism of sulfur component monocarboxylic acid and the biosynthesis of glutathione derivatives. Of note, SOX9 was overexpressed in the livers of PSC patients with the worst clinical prognostic profiles (cohort 2). A distinct quantification of SOX9 was found during a comparison of hepatic stem/progenitor cells between primary sclerosing and PBC.^34^ On the NR0B2 cell trajectory of Abcb4−/− cholangiocytes, some molecules implicated in carcinogenesis were evidenced: GSTA3, ID2, and TMEM45A, which is a transmembrane molecule considered to be oncogenic in several cancers.

GSTA3 belongs to the glutathione S-transferase (GST) α-class, which converts lipid peroxides into glutathione conjugates^35^ and was found with a pseudotime expression similar to NR0B2 in cholangiocytes indicating their closed regulation during this cell trajectory. GSTA3 plays a role in inhibiting hepatic stellate cell activation and GSTA3 KO mice display liver injury, oval cell proliferation, and cholangiocarcinogenesis.^36^ The ID2 is a member of the basic helix-loop-helix transcription factor family. Similar to other ID proteins, ID2 lacks a DNA-binding domain, so its targets for transcription of the downstream cell cycle regulators include p16 or p21.^37^ And similar to ID1 and ID3 proteins, ID2 is overexpressed in biliary tract cancer. ID protein expression is also deregulated in many tumors, including pancreatic cancer which is a malignancy that is related to bile tract cancers to some degree. In the case of these cancers, and in a subgroup of patients who had received chemotherapy, if the nuclear expression of ID2 was negative, they presented with a better overall survival than those in whom this nuclear expression was positive.^38^ TMEM45A is a transmembrane protein that is overexpressed in many cancers and supposed to be an oncogene^39^, ^40^, ^41^, ^42^, ^43^, ^44^ but is unknown in the carcinogenesis of cholangiocarcinoma.

NRs are ligand-activated transcription factors which control a broad spectrum of genes involved in BA metabolism, inflammation, cell proliferation, and tissue repair (including fibrosis) which renders them highly attractive targets for the treatment of metabolic and cholestatic disorders.^28^ The NR0B2 NR participates in the deregulation of BA metabolism homeostasis which is probably an early step in the pathogenesis of PSC that occurs before the stages of inflammation and fibrosis. In PSC livers, an early independent hepatic deregulation of NR0B2 highlighted a metabolic and premalignant reprogramming of the cholangiocyte cell type at a single-cell level. NR0B2 is a BA metabolism regulator and is the partner of FXR which is a therapeutic target of obeticholic acid. The therapeutic targeting of NR0B2 may potentiate that of FXR and thus enable action on early events of the disease and prevention of its progression.

## Methods

### Biomedical PubMed Text Mining

“Génie” data-mining tools were able to perform gene prioritization^9^ by linking scientific literature from the MEDLINE database with gene information from the NCBI Gene database and orthologous gene information on different species from the HomoloGene database.^12^^,^^45^ The Génie algorithm works by connecting the gene identifier and gene ortholog NCBI databases with genes related to the keywords in article abstracts from the PubMed literature resource. PSC-related symptoms Medical Subject Heading terms were individually submitted to the “Génie” algorithm on July 2, 2021, as keywords. All PubMed abstracts were taken as the background for the analysis of genes corresponding to *Homo sapiens* coding proteins. Information on all orthologs was collected during the process. The *P* value cutoff point for abstract selection was fixed at *P* < .01, and the FDR cutoff point for gene selection was fixed at *P* < .01.

### Public Data Sets

#### Transcriptome Studies

GSE61256 is a data set of human liver biopsies (n = 134) that was developed to study nonalcoholic steatohepatitis. Among the controls, we found 14 patients with a diagnosis of PSC with very few clinical annotations regarding severity.^7^ The transcriptomes were downloaded and annotated with the corresponding platform (Gene Expression Omnibus Platform identifier for annotation: GPL11532) used for the Affymetrix GeneChip Human Gene 1.1 ST Array (alias HuGene-1_1-st) available at the following address: https://www.ncbi.nlm.nih.gov/geo/query/acc.cgi?acc=GPL11532 (accessed on July 2, 2021).

GSE54557 is a data set on livers from *Mus musculus*, either KO for FXR (Cre recombinase on C57BL/6J genetic background) or receiving the FXR agonist.^16^ FXR-null mice harbor inhibition of CYP7A1 transcription and of BA synthesis. When challenged with a diet containing cholic acid, FXR-null mice experienced severe hepatotoxicity and wasting when compared to wild-type mice.^18^ Each condition was tested in triplicate in mice aged 10–16 weeks. The transcriptome matrix was downloaded and annotated with the GPL8321 platform corresponding to the DNAchip technology, Affymetrix Mouse Genome 430A 2.0 Array (alias Mouse430A_2) available at the following address: https://www.ncbi.nlm.nih.gov/geo/query/acc.cgi?acc=GPL8321 (accessed on July 2, 2021).

An independent cohort of PSC patients with clinical annotations was developed. The liver transcriptome had been obtained by the RNA sequencing of liver tissue from patients with PSC (n = 74).^6^ In this validation cohort, the expression of predictive molecular markers was evaluated according to clinical assessments involving events such as ascites, spontaneous bacterial peritonitis, variceal hemorrhage, hepatic encephalopathy, ascending cholangitis, cholangiocarcinoma, hepatocellular carcinoma, liver transplantation, and death. The expression of biomarkers was also stratified using Ishak staging: mild (0–2), moderate (3–4), and cirrhosis (5–6).

GSE168758 *Mus musculus* Abcb4−/− single-cell transcriptome liver samples^22^ came from mice lacking the phospholipid floppase Mdr2 (Abcb4) with a PSC phenotype. The bile ducts of Mdr2(−/−) mice displayed disrupted tight junctions and basement membranes, BA leakage into portal tracts, the induction of a portal inflammatory (CD11b, CD4-positive) infiltrate, and the activation of proinflammatory (tumor necrosis factor-alpha, interleukin-1β) and profibrogenic cytokines (transforming growth factor-1β). This resulted in the activation of periductal myofibroblasts leading to periductal fibrosis, separating the peribiliary plexus from bile duct epithelial cells and ultimately causing atrophy and death of the bile duct epithelium.^19^ Single-cell transcriptome libraries of liver cells were prepared using the Chromium Single Cell 3′ NextGEM Reagent Kit v3.1 (10× Genomics) in order to perform sequencing on NextSeq 550 (Illumina) and demultiplexing the pipeline with CellRanger v5.0.0 (10x Genomics). MTX matrix file formats were downloaded at https://www.ncbi.nlm.nih.gov/geo/query/acc.cgi?acc=GSM5165876 (accessed on July 2, 2021).

#### Bioinformatics Analysis

Bioinformatics analysis was performed in the R software environment (version 4.1.0) under Ubuntu version 20.04 LTS. Graphic representations were generated with the ggplot2 graph definition implemented under the ggplot2 R-package version 3.3.5.

#### Transcriptome Analyses

An unsupervised analysis was performed using principal component analysis with FactoMineR R-package version 2.4.^46^ Gene functional enrichment analyses were performed with the ToppGene web application.^47^ Differentially expressed genes were identified using the Linear Model from Microarray algorithm implemented under the R Bioconductor package version 3.44.3.^48^ FXR regulation dependency was analyzed using the Pavlidis Template Matching algorithm. This analysis retained significant genes based on their absolute correlation coefficient values and *P* values lower than .05.^17^ Expression heatmaps were drawn using the pheatmap R-package version 1.012.

#### Single-Cell Transcriptome Analysis

Single-cell transcriptome analyses at the whole liver level were performed using the Seurat4 R-package version 4.0.3.^49^ After canonical correlation, filtration (cells expressing a minimum of 300 genes), and scaling between replicated samples (n = 4) of the WT and Abcb4−/− groups, a normalized single-cell object comprising 46,087 transcriptomes was built. Dimension reduction was achieved using principal component analysis on the most variable features (50 PCA components during dimension reduction). Then t-distributed stochastic neighbor embedding and Uniform Manifold Approximation and Projection dimensionality reductions were performed on the 30 best components of the PCA. Cell cluster communities were detected through graph-based clustering approaches.^50^ Abcb4−/− cholangiocytes identified in clusters C0-C5-C11 were selected and randomly downsampled to 10% of their initial quantity in the data set. The resulting matrix containing 1316 cholangiocytes was processed to perform downstream analyses. Single-cell trajectories were then determined on Abcb4−/− cholangiocytes using the monocle2 R Bioconductor package version 2.20.0.^51^ Cell hierarchy was constructed on the alternative expression of 2 distinct cluster markers in Abcb4−/− cholangiocytes. Pseudotime transformation on cell trajectories was performed with the DDRtree algorithm, and pseudotime cluster identification was achieved through a pseudotime expression heatmap drawn using the best genes found to be significant.

#### Network Analyses

Gene and function relationships identified during the representative functional analysis were collected, and a network was built using Cytoscape standalone software version 3.6.0^52^ to enable their visualization. PPI networks were built with NetworkAnalyst^53^ web tools based on the STRING interaction network^54^ and liver-specific DifferentialNet databases. Functional inference was performed on the PPI networks using the Gene Ontology Biological Process database.^55^

## Supplementary Materials

Tables A1–A4 and Figures A1–A4
