# Supplementary material for: Early Deregulation of Cholangiocyte NR0B2 During Primary Sclerosing Cholangitis
Source: Gastro Hep Adv. 2022 Aug 13;2(1):49–62. doi: 10.1016/j.gastha.2022.07.023 (PMC11307415; doi:10.1016/j.gastha.2022.07.023)
Supplement: Tables A1–A4 and Figures A1–A4 [file mmc1.docx]

SUPPLEMENTAL MATERIALS

**Supplemental Figures**

**Supplemental Figure 1: Stratification of PSC and PBC liver transcriptomes using unsupervised analysis:** Second map of principal component analysis performed on liver transcriptomes from the GSE61260 dataset with PCS-related genes found by text mining.

**Supplemental Figure 2: Single cell heterogeneity of clusters identified in WT and Abcb4-/- livers:** A/ whole liver UMAP dimension reduction with a summary of cell cluster identifications; B/ Cldn7 featureplot which identified cholangiocyte cell subgroup; C/ Apoc4 featureplot which identified hepatocyte cell subgroup; D/ Kdr featureplot which identified endothelial cell subgroup; E/ Cd68 FeaturePlot which identified the macrophage cell subgroup; F/ Cd8a FeaturePlot which identified the T-lymphocyte cell subgroup; G/ Ms4a1 Kdr FeaturePlot which identified the B-lymphocyte cell subgroup.

**Supplemental Figure 3: Single cell differential expression of Sox9, Nr0b2 and Epcam in cholangiocyte clusters of WT and Abcb4-/- livers:** A/ Violinplot of Sox9 expression in cholangiocyte clusters 0-5-11 stratified on phenotype wildtype versus Abcb4-/-; B/ Violinplot of Nr0b2 expression in cholangiocyte clusters 0-5-11 stratified on phenotype wildtype versus Abcb4-/-; C/ Violinplot of Epcam expression in cholangiocyte clusters 0-5-11 stratified on phenotype wildtype versus Abcb4-/-.

**Supplemental Figure 4: Pseudotime transformation of the cholangiocyte cell trajectory in Abcb4-/- livers:** A/t-SNE plot of Abcb4-/- cholangiocytes with group stratification based on the alternative expression of Sox9 and Nr0b2; B/ t-SNE plot of Abcb4-/- cholangiocytes with group stratification identified in Seurat clustering: clusters 0-5-11; C/ pseudotime tree with group stratification identified in Seurat clustering: clusters 0-5-11; D/ pseudotime tree with expression of sox9 as dot size and group stratification as dot color; E/ pseudotime tree with expression of Nr0b2 as dot size and group stratification as dot color; F/ pseudotime expression plot of markers found to be closely regulated with Nr0b2 on the pseudotime cell trajectory.

**Access to Data:**

Supplemental tables are accessible online through Mendeley dataset:

Desterke, Christophe; Feray, Cyrille (2022), “NR0B2 regulation during Primary Sclerosing Cholangitis defines a metabolic and pre-malignant reprogramming of Cholangiocyte”, Mendeley Data, V1, doi: 10.17632/jcpp3ksm5m.1

<https://data.mendeley.com/datasets/jcpp3ksm5m/1>

**Supplemental Tables**

**Supplemental Table 1. Text mining of the 525 ranked genes found in PUBMED with symptom keywords:** this Table describes the 525 ranked genes obtained by text mining with the ‘Génie’ algorithm against the three MESH terms: biliary inflammation, biliary fibrosis and biliary stasis. The respective ranks, p-values and PMID identifiers collected for each gene and each MESH term are presented.

**Supplemental Table 2. Symptom-related genes found to be differentially expressed in livers from primary sclerosing cholangitis patients:** Differentially expressed gene analysis results (logarithmic Fold Change, Average Expression and Adjusted p-values) are presented with respective machine learning predictive scores for supervised sample categories from the GSE61256 dataset (PSC versus other liver samples).

**Supplemental Table 3: Pavlidis Template Matching for FXR dependency in the liver transcriptome.** Results of Pavlidis template analysis used to describe the FXR regulation dependency of PSC genes in liver samples from the GSE54557 dataset. For each gene, the R-Pearson correlation coefficient and its respective p-value are presented in this Table.

**Supplemental Table 4. Best one hundred genes found to be significant in the pseudotime trajectory of Abcb4-/- cholangiocytes.** Best one hundred ranked genes found to be significant on the pseudotime cell trajectory of Abcb4-/- cholangiocytes based on the alternative expression of Nr0b2 and Sox9 in GSE168758.
